# Urethane-Acrylate/Aramid Nanocomposites Based on Graphenic Materials. A Comparative Study of Their Mechanical Properties

**DOI:** 10.3390/polym12102388

**Published:** 2020-10-16

**Authors:** Israel Gago, Manuel del Río, Gerardo León, Beatriz Miguel

**Affiliations:** Departamento de Ingeniería Química y Ambiental, Universidad Politécnica de Cartagena, Paseo Alfonso XIII, 30203 Cartagena, Spain; israel.gago@upct.es (I.G.); manuel.delrio@edu.upct.es (M.d.R.); beatriz.miguel@upct.es (B.M.)

**Keywords:** graphenic materials, graphene nanocomposites, urethane-acrylate/aramid, mechanical properties

## Abstract

Urethane-acrylate thermoset resins (UATR) are a new type of polymeric matrix that have recently made a strong breakthrough in the composites sector. This is because of their properties, which make them an advantageous alternative to epoxy resins, especially if they are reinforced with high-performance fibers such as aramids. Graphene-based nanocomposites are one of the most dynamic research fields in nanotechnology, because graphenic materials greatly improve the properties of traditional composites. This work represents a comparative study of the effect of adding three types of graphenic materials on the mechanical properties of UATR/aramid composites. Several UATR polymeric matrices were doped at 2% *w*/*w* with graphene nanoplatelets (GNPs), reduced graphene oxide (rGO) and pristine few-layer graphene (FLG), and reinforced with Twaron CT709 para-aramid fibers. The obtained laminates showed low density (1.38 g·cm^−3^), a high volumetric fiber–resin ratio (80:20), homogeneous dispersion of the nanoreinforcement, high reproducibility, and easy scalability. The tensile, flexural and impact strength properties of the undoped composite and the graphene-doped nanocomposites were determined. FLG-doped nanocomposites showed the highest increase in all the mentioned mechanical properties and attained a very significant relative improvement over the undoped laminate (up to 134.4% in a_CU_).

## 1. Introduction

Composites are a very important family of materials used in a wide variety of high-performance structural applications due to their excellent mechanical properties, low density, and resistance to environmental conditions and chemical agents [1]. Carbon fiber/epoxy composites are the most widely used in these sectors because they are very light and can effectively withstand isostatic stresses, to the point that they are replacing their metal counterparts in many cases. However, they also have drawbacks, such as their low capacity to absorb the energy of an impact, the need for complex curing and post-curing processes, and the high environmental impact of the epoxy thermoset resins [2].

Recently, a new type of polymeric matrix has burst into the high-performance composite materials sector: urethane-acrylate thermoset resins (UATR). UATRs are a very interesting alternative to epoxy resins, because they have very good mechanical properties, they do not require high-temperature curing processes, and they are less damaging to the environment than epoxy resins. By combining a UATR matrix with high-performance reinforcement fibers, such as para-aramids (poly-4-phenyleneterephthalamide), it is possible to achieve light composite materials with excellent mechanical properties for structural applications, while maintaining good fracture toughness (impact strength), which is a notable value-added feature for high-performance engineering applications [3].

Since its discovery in 2004, graphene has been regarded as a promising material for new applications in the field of nanocomposites due to its excellent properties, which include a surprising strength-to-weight ratio, extremely high fracture toughness, and its capability to improve the mechanical properties of several polymers and composites [4,5,6,7,8,9,10]. In recent years, several studies have shown that the addition of graphene to carbon fiber/epoxy composites can improve their mechanical properties due to the ability of graphene to improve the interfacial cohesion between the matrix and the reinforcement fibers [11,12]. However, because of the recent development of UATR resins, there are no data available on how graphenic nanoreinforcements can modify the mechanical properties of nanocomposites based on this new type of polymer matrix.

This paper presents initial results concerning the manufacture of novel nanocomposites based on graphene-doped UATR/aramid, and their most relevant mechanical properties. Nanocomposites with a UATR matrix doped with three different types of graphenic nanomaterials and reinforced with para-aramid fibers were prepared. Graphene nanoplates, reduced graphene oxide and pristine few-layer graphene, at a 2% doping ratio (*w*/*w*), were used as nanoreinforcements, and the influence of each graphenic nanomaterial on the undoped UATR/aramid composite was analyzed by determining their respective tensile, flexural and impact properties.

## 2. Materials and Methods

### 2.1. Materials

**Figure 1:**
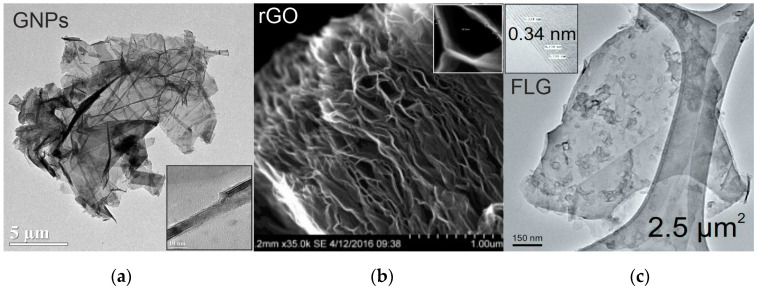
Electronic micrographs of the graphenic nanomaterials used: (**a**) GNPs (graphene nanoplatelets); (**b**) rGO (reduced graphene oxide); (**c**) FLG (pristine few-layer graphene). Enlargements inserted in the corresponding pictures show the number of layers and the distance between crystalline planes.

GNPs (graphene nanoplatelets) (GNNP0051) were purchased from ACS Materials^®^ (Pasadena, CA, USA), rGO (reduced graphene oxide) (R-15-P/S-106) was purchased from Abalonix^®^ (Oslo, Norway) and FLG (pristine few-layer graphene) was produced in our labs. Figure 1 includes electronic micrographs showing the morphological characteristics of the graphenic materials used, and Table 1 includes their main characteristics.

Crestapol 1260^®^, a commercially available UATR of high toughness and low viscosity (Table 2), is specially designed for the manufacture of high-performance composite parts. In our case, it was purchased from Scott Bader^®^ (Wellingborough, UK) and used as the thermoset resin matrix.

Twaron CT709^®^, a commercial plain weave fabric (bidirectional 0°/90°, 50:50 warp/weft ratio, 200 g·m^−2^) made of high tenacity para-aramid microfilament yarns and specially designed for the manufacture of high-performance ballistic protection systems, was purchased from Teijin Aramid^®^ (Arnhem, The Netherlands) and used as reinforcing fibers.

### 2.2. Methods

**Figure 2:**
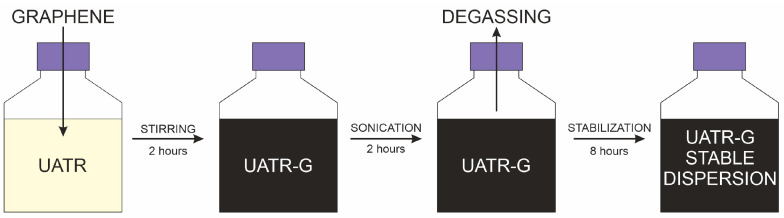
Schematic representation of the preparation procedure for graphene-doped UATR (UATR-G) matrices.

The graphene-doped UATR/aramid nanocomposites were prepared at room temperature, following a similar procedure to that described in previous works [3,13,14]. Graphenic nanomaterials were added to the liquid UATR matrix (2% *w*/*w*) and mechanically stirred at 800 rpm for 2 h in a screw-top flask. Graphene-doped UATR (UATR-G) was then sonicated in an ultrasonic bath (50–60 Hz) for 2 h, with the cap opened every 30 min to allow degassing, in order to prevent the formation of air bubbles in the final laminate. Once the sonification process was completed, the UATR-G dispersion was left overnight for colloidal stabilization and protected from sunlight, to prevent degradation of the polymer matrix caused by undesired photochemical reactions. No coupling or dispersing agent was used in the preparation of the matrices so as not to alter the chemistry of the UATR polymer or the possible interactions of the graphenic nanomaterials among themselves (self-aggregation) or with the rest of the components of the final nanocomposite. To ensure the homogeneity of the laminates, the matrix used for the undoped laminate was subjected to the same procedure as the UATR-Gs but without the addition of any doping agent. Figure 2 provides a schematic representation of the preparation process for the UATR-G matrices.

**Figure 3:**
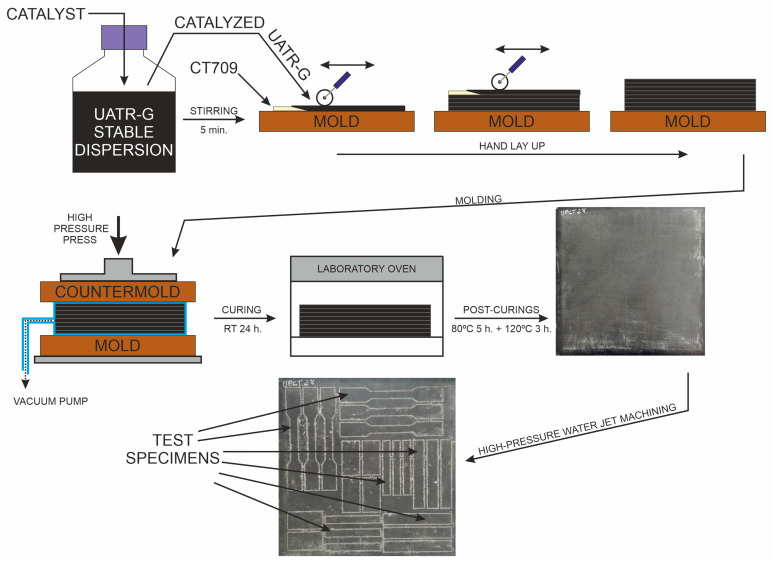
Schematic representation of the preparation procedure for UATR-G/aramid nanocomposites. RT = Room Temperature.

The catalyst (Trigonox 239, Cumyl hydroperoxide) was added to the stabilized UATR-G colloidal dispersion (2% *w*/*w*) with mechanical stirring at 250 rpm for 5 min. Laminates, reinforced with Twaron CT709 para-aramid fibers, were made by means of a combined technique of hand lay-up and vacuum-assisted molding under compression at room temperature. Once the lamination process was completed, the laminates were cured for 24 h at room temperature, followed by two post-curing steps at 80 °C (5 h) and 120 °C (3 h), according to the manufacturer’s technical specifications. After curing, the corresponding specimens were machined from the laminated plates by high-pressure water jet cutting, for the characterization of their mechanical properties. Figure 3 shows a schematic representation of the manufacturing process.

Tensile modulus (E_t_, GPa) and tensile strength (σ_t_, MPa) were characterized in accordance with the ISO 527-4: 1997 standard by using Microtest EM2/300/FR apparatus. Flexural strength (σ_f_, MPa) and flexural modulus (E_f_, GPa) were characterized in accordance with the ISO 14125: 1998 standard by using MicrotestEM2/300/FR apparatus. Charpy impact strength (a_cU_, kJ·m^−2^) was characterized according to the ISO 179-1:2010 standard by using a Zwick I 5113.100 Charpy pendulum for composite materials. Table 3 shows the dimensions of the specimens, and the number of specimens tested.

## 3. Results and Discussion

### 3.1. Laminated Plates

**Figure 4:**
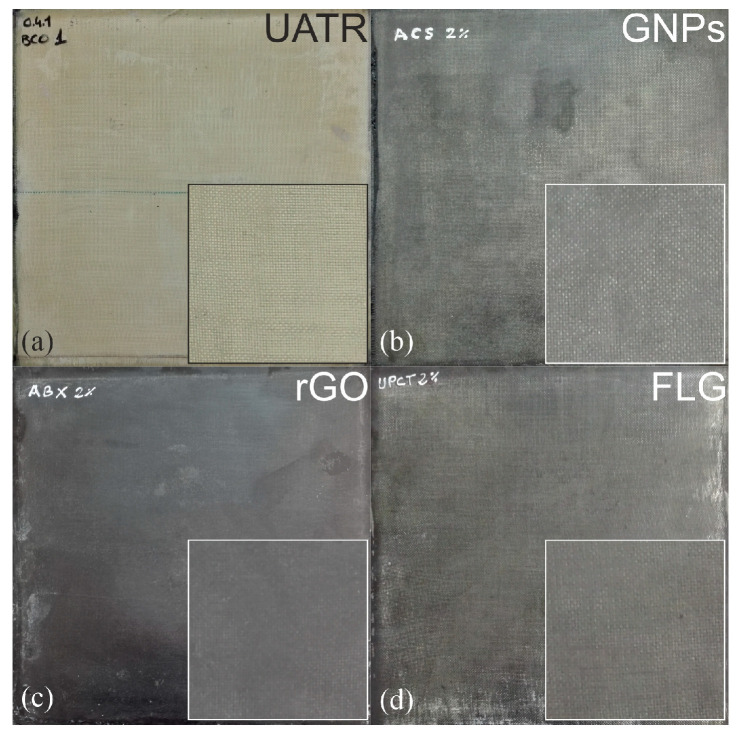
(**a**) Undoped UATR/Aramid composite laminate; (**b**) UATR-GNPs/Aramid nanocomposite laminate; (**c**) UATR-rGO/Aramid nanocomposite laminate; (**d**) UATR-FLG/Aramid nanocomposite laminate. Magnifications (×4) are inserted as a smaller square in each picture.

Figure 4 shows the different laminated plates manufactured. The good quality of the laminates is evident in all cases, with no detectable bubble formation [15,16] and excellent dispersion in the matrix of the different graphenic materials, regardless of their different number of layers, specific surface area, density of defects in the crystal lattice or absence/presence of functional groups.

It is important to note that the homogeneous dispersion of the graphenic materials within the nanocomposite is essential in order to ensure the properties of the final nanocomposites are evaluated correctly. The specimens were 0.5 cm thick, with an 80:20 fiber/matrix ratio by volume (*v*/*v*) in all cases. The final density of the obtained nanocomposites was, in all cases, 1.38 g·cm^−3^, which is an improvement (or lightening) of 28.8% compared with a typical epoxy/fiberglass composite laminate (1.79 g·cm^−3^), and 11.5% compared with a typical epoxy/carbon fiber composite laminate (1.55 g·cm^−3^). This weight saving compared to traditionally used composites implies a significant improvement in potential applications in the aerospace and defense sectors, where the use of lightweight and high-performance materials is imperative.

### 3.2. Mechanical Properties

**Figure 5:**
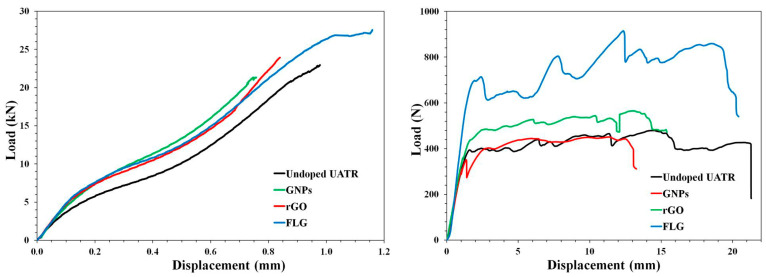
Force–displacement curves for tensile (**left**) and flexural (**right**) tests.

Figure 5 shows the force–displacement curves for the tensile and flexural tests carried out.

**Figure 6:**
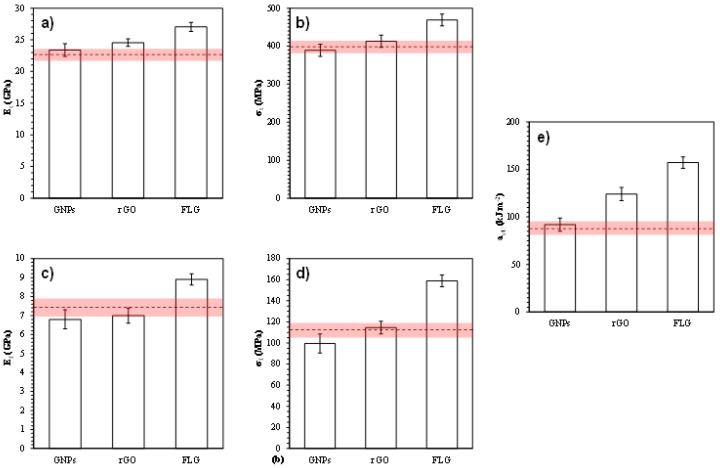
Mechanical properties of the tested laminated plates: (**a**) Tensile modulus (E_t_); (**b**) Ultimate tensile strength (σ_t_); (**c**) Flexural modulus (E_f_); (**d**) Ultimate flexural strength (σ_f_); (**e**) Charpy impact strength (a_cU_). The dashed line represents the mean value obtained for an undoped UATR/Aramid composite with its corresponding error interval (light red band).

Figure 6 shows the results obtained for the mechanical properties of the tested laminated plates: (a) tensile modulus (E_t_); (b) ultimate tensile strength (σ_t_); (c) flexural modulus (E_f_); (d) ultimate flexural strength (σ_f_); (e) Charpy impact strength (a_cU_). In all cases, the error obtained for all measurements was less than 5%, as required by the corresponding standard.

GNPs do not show any significant improvement over the mechanical properties of the UATR-GNPs/aramid nanocomposite. As suggested in previous studies carried out with epoxy resin matrices, this may be due to the greater lateral size and number of layers in GNPs, which significantly reduces the effectiveness of graphenic material as a nanoreinforcing agent [17]. The large lateral size of the GNPs provides them with a natural tendency to wrinkle and fold back on themselves because of thermodynamic instabilities in the crystal lattice, causing a drastic reduction in their specific surface area while making it difficult for them to be effectively impregnated by the resin matrix [18]. The high number of layers in GNPs also has a negative effect on their reinforcing capability, since the mechanical properties of graphenic materials decrease rapidly above 10 layers [19]. Consequently, GNPs cannot be considered as an appropriate nanoreinforcement for UATR/Aramid composites.

Compared with the corresponding values of the undoped UATR/Aramid laminates, UATR-rGO/aramid nanocomposites do not show significant changes in tensile and flexural properties, although their Charpy impact strength is considerably (42.5%) higher. This increase may be related to the high surface area of rGO [20]. However, that increase is much lower than that in FLG (80.5%) due to the higher surface area of FLG [21] and to the lower ability of rGO nanocomposite to transmit efforts due to its layered structure (Figure 1).

The FLG-doped nanocomposite (UATR-FLG/aramid) showed important improvements in all the studied mechanical properties, compared with the corresponding values of the undoped UATR/aramid laminate: an increase in tensile properties (19.2% for Et and 17.4% for σ_t_), in flexural properties (20.3% for Ef and 41.2% for σf), and in Charpy impact strength (80.5%). This may be explained if we take into account that FLG has the best quality of the three graphenic materials studied, since it has the lowest number of layers (≤7), the best mechanical properties (E = 1.0 TPa, σ = 130 GPa), the lowest surface density of defects in the crystalline lattice, and the highest specific surface area, aspect ratio and ability to interact with the other nanocomposite components at a nanoscale [22]. The effectiveness of FLG as a nanoreinforcement can be explained as a result of its stress distribution, ductilization, crack mitigation, interfacial interactions and fracture toughness properties [23].

The excellent mechanical properties of pristine graphene, together with its bidimensional morphology and large specific surface area, make it a very effective nanoreinforcement, since it can transfer part of its mechanical properties to the matrix and distribute the stresses to which it is subjected. This increases the capacity of the nanocomposite to withstand local stresses and drastically reduces stress concentrations within the matrix, which results in a significant increase in the mechanical properties of the nanocomposite [24,25,26,27].

The sp^2^ hybridization of the C–C bonds of graphene gives it a high degree of atomic packing in its crystalline lattice and its bidimensional morphology. This means that when a small amount of graphene is dispersed homogeneously in a thermosetting polymer, the amount of cross-linking that the matrix can form during its curing process is slightly reduced, resulting in a reduction in its typical brittle fracture behavior due to a ductilization process [28].

The two aspects described above are also involved in delaying the nucleation of cracks within the material, since the addition of FLG gives the resulting nanocomposite greater stiffness, strength, and ductility. In addition, once a crack forms and begins to grow, the nearest FLG flakes, which are randomly oriented, disperse the stress concentrations that form around the advance tip in many different directions, drastically reducing its ability to propagate due to the increased energy that is needed for the crack to continue growing [29]. If the stress applied to the material continues to grow, the crack will advance until it encounters a flake of graphene oriented almost perpendicularly to its growth vector. In this situation, and given that the mechanical properties of the FLG are much greater than those of the matrix, in order for the crack to continue to progress it must branch into two or more complex pathways. This results in an even greater dissipation of energy through the material, which normally causes the growth process to stop and thus, a significant delay in crack coalescence phenomena. Since the matrix is the component responsible for the transmission of stresses to the reinforcing fibers, the delay in the nucleation, growth and coalescence of the cracks means that such stress transmission becomes more effective and durable, which in turn produces a generalized increase in the mechanical properties of the nanocomposite as a whole [29,30].

The strength of the micromechanical interactions at the interface between the individual components plays a very important role in the final physical properties of a composite. Due to their properties, the graphene flakes dispersed within the nanocomposite effectively act as anchor points, significantly increasing the micromechanical interactions at the fiber/matrix interface, which increases their strength and expands their range [29,30]. This increases the number of fibers and the interfacial area that contribute effectively to support the applied load, which is distributed more homogeneously throughout the material, delaying the rupture of the fiber/matrix interfacial junction. This mechanism is related to the three mechanisms mentioned above and acts with them synergically, notably increasing the mechanical properties of the final nanocomposite [31].

One of the most relevant properties of graphene for its application in engineering solutions is its high fracture toughness. The very significant improvement observed in the Charpy impact strength of the UATR-FLG/aramid nanocomposite can be partially explained by the mechanisms set out for isostatic mechanical properties (tensile and flexural). However, another consideration that is directly related to the characteristic high fracture toughness of FLG should be mentioned: pristine graphene presents brittle fracture mechanics and its critical stress intensity fracture factor (Kc) was found to be 4.0 MPa·m^−1/2^, more than six times higher than the average value of epoxy resins (0.65 MPa·m^−1/2^) [32]. Many papers mention that the nanoreinforcing effect of graphene in a polymer matrix becomes more pronounced, as the difference between the value of a given mechanical property in the nanoreinforcement and in the polymer also becomes more pronounced, with improved fracture toughness being one of the properties in which this nanoreinforcing effect is most evident [23,28,33,34,35].

## 4. Conclusions

The ability of three different types of graphenic materials (GNPs, rGO and FLG) to improve the tensile, flexural and Charpy impact mechanical properties of a composite, formed by a thermoset urethane-acrylate polymer matrix reinforced with para-aramids has been studied. For this, the experimental data obtained by testing undoped samples of a UATR/aramid composite and those of several UATR-G/aramid nanocomposites individually doped with GNPs, rGO or FLG at 2% (*w*/*w*) were compared.

GNPs did not produce any significant improvement in the mechanical properties of the UATR-GNPs/aramid nanocomposites and, consequently, GNPs cannot be considered as a suitable nanoreinforcement. In the case of rGO, UATR-rGO/aramid nanocomposites showed no significant changes in tensile or flexural properties but did show a notable increase in acU (42.5%). With regards to FLG, the UATR-FLG/aramid nanocomposite showed important improvements in all the considered mechanical properties: 19.2% in tensile modulus; 17.4% in ultimate tensile strength; 20.3% in flexural modulus; 41.2% in ultimate flexural strength; and 80.5% in Charpy impact strength. Therefore, FLG can be regarded as the best nanoreinforcing agent to improve the mechanical properties of UATR/aramid composites.

## Figures and Tables

**Table 1:** Main characteristics of the three graphenic nanomaterials used.

Property	GNPs	rGO	FLG
Chemical composition: Carbon (C) Oxygen (O) Impurities (I)	C > 99%	C 81%; O 17%; I 2%	C > 99%
C/O atomic ratio	Na	6.5	Na
Average layers (sheets type)	30 (isolated sheets)	8 (in sheet stacks)	7 (isolated sheets)
D_50_	7 µm	Na	1 µm
Specific Surface Area	40 m^2^·g^−1^	400 m^2^·g^−1^	1000 m^2^·g^−1^
Functional groups	-	-OH; -COOH; -O-	-

**Table 2:** Fundamental properties of the urethane-acrylate thermoset resins (UATR) (according to the manufacturer’s datasheet).

Density (25 °C)	Viscosity (25 °C)	Barcol Hardness	Ultimate Tensile Strength	Tensile Modulus
1.04 g·cm^−3^	2 poise	38 a.u.	76 MPa	3.28 GPa

**Table 3:** Specimen dimensions and number of specimens tested.

Property	Specimen Dimensions			Specimens Tested
	Length (mm)	Width (mm)	Thickness (mm)	
Tensile (E_t_, σ_t_)	150	10	5	20
Flexural (E_t_, σ_t_)	100	15	5	20
Charpy impact (a_CU_)	80	10	5	20
